# Structural effect of two-dimensional BNNS on grain growth suppressing behaviors in Al-matrix nanocomposites

**DOI:** 10.1038/s41598-018-20150-5

**Published:** 2018-01-25

**Authors:** Seungjin Nam, Kunok Chang, Woonki Lee, Moon J. Kim, Jun Yeon Hwang, Hyunjoo Choi

**Affiliations:** 1 School of Advanced Materials Engineering, Kookmin University, Seoul, 02707 Republic of Korea; 2 Nuclear Materials Safety Research Division, Korea Atomic Energy Research Institute, Daejeon, 34057 Republic of Korea; 3 Institute of Advanced Composite Materials, Korea Institute of Science and Technology, Jeonbuk, 55324 Republic of Korea; 4 Department of Materials Science and Engineering, The University of Texas at Dallas, Richardson, TX 75080 USA

## Abstract

While nanocrystalline (NC) metals exhibit superior strength to conventional microcrystalline metals, their thermal instability has hampered their application at high temperatures. Herein, two-dimensional (2D) boron nitride nanosheets (BNNS) are proposed as reinforcement to enhance the strength as well as the thermal stability of NC Al. The strength of pure Al was increased from 80 to 468 MPa by refining its grains from ~600 to ~40 nm, and it was further enhanced to 685 MPa by incorporating 2 vol% of BNNS. Moreover, the small amount of BNNS was found to effectively suppress grain growth of NC Al at 580 °C (~0.9 T_m_, where T_m_ is the melting point of Al), which prevented a strength drop at high temperature. Finally, the Zener pinning model in conjunction with phase-field simulations was utilized to qualitatively analyze the effect of the BNNS on the grain boundary pinning as a function of volume, shape, and orientation of the reinforcement. The model demonstrated that the pinning force of 2D reinforcements is much higher than that of spherical particles. Hence, 2D BNNS offer the possibility of developing Al-matrix nanocomposites for high-temperature structural applications.

## Introduction

According to the Hall–Petch relationship^1,2^, a reduction in grain size (*D*) results in an increase in the yield strength with a *D*^−1/2^ dependency; when the grain size is relatively large, greater stress can be concentrated near the adjacent grains due to the pile-up of multiple dislocations, leading to decreased yield strength. When grain size reduces to within the nanocrystalline (NC, grain sizes below 100 nm) regime, however, the activities of lattice dislocations become less significant and the yield stress starts to deviate from the Hall–Petch relationship^3–7^. For example, Al shows a dramatic increase in yield strength with reduced *D* in this regime, following *D*^−1^ dependency (perfect dislocation model^8^) instead of *D*^−1/2^ dependency (the Hall–Petch relationship)^9^. Hence, grain refinement in the NC regime can be a breakthrough toward enhancing the strength of Al, which currently ranks second as the most frequently used metal, behind steel, due to its economical and lightweight properties. Applications of NC metals, however, are restricted owing to their poor thermal stability at high temperatures. Migration of the grain boundary is largely driven by the high enthalpy in large areas of the grain boundaries, as well as their local curvature in NC metals. In particular, grain growth in Al occurs rapidly because of its low melting temperature (993 K) and its low activation energy for grain boundary self-diffusion (84 kJ/mol)^10^, compared to other metals (Supplementary Table 1). Hence, the suppression of grain growth in NC Al at high temperatures is attracting significant interest.

Grain growth can be suppressed by reducing the driving force of grain boundary migration (thermodynamic stabilization) and/or by increasing the activation energy for grain boundary migration (kinetic stabilization)^11–14^. Under a given thermomechanical environment, the driving force for grain boundary migration can be controlled by manipulating the intrinsic microstructural features, especially the grain boundary energy (${\gamma }_{{\rm{GB}}}$)^15,16^. Segregation of solute atoms at the grain boundaries is an effective method of reducing grain boundary energy, as illustrated by the following equation:

1 $${\gamma }_{{\rm{GB}}}={\gamma }_{0}-{\rm{\Gamma }}({\rm{RTlnX}}+{\rm{\Delta }}{H}_{seg})$$

where *γ*_0_ is the boundary energy for pure solvent, *Γ* is the solute excess of the grain boundary, *R* is the ideal gas constant, *T* is the temperature, *X* is the solute composition, and Δ*H*_*seg*_ is the segregation enthalpy of solute atoms to the grain boundary^17^. A proper alloying element can be selected by considering the solubility of the solute atoms and strain energy through lattice mismatching, as well as the inherent chemical and interfacial energies^18–22^. For Al alloys, Mn, Pb, Sr, Yb, and Zr have been experimentally and/or computationally demonstrated to effectively suppress grain growth by reducing the segregation enthalpy^23–26^.

By comparison, kinetic stabilization operates according to the ideal grain growth kinetic model during isothermal heat treatment, as expressed in the following equation:

2 $${D}_{t}^{n}-{D}_{0}^{n}={k}_{gg}t$$

where *D*_0_ and *D*_t_ are the average grain sizes before and after heat treatment for a period of time (*t*), respectively, *n* is the grain growth exponent, and *k* is a kinetic constant representing grain boundary mobility. *k* is expressed as a function of an Arrhenius-type equation $({k}_{{\rm{gg}}}={k}_{0}\exp (-\frac{{Q}_{{\rm{gg}}}}{RT})$, where *k*_0_ is the pre-exponential term and *Q*_gg_ is the activation energy for grain boundary migration). In general, the grain growth exponent is assumed to be 2 for pure materials at very high temperatures and increases with decreasing heat treatment temperatures^27^. The activation energy for grain boundary migration increases when the boundary is hindered or dragged by pores, solute atoms (impurities), precipitates, and chemical ordering^28–31^. For the case of solute atoms (impurities), the drag effect on grain boundary migration is influenced by the solute diffusivity within the lattice, the concentration of solute atoms at the grain boundaries, and the size mismatch between the solvent and solute atoms^32–34^. Even though the drag effect of solute atoms increases with increasing concentration, it is restricted by the solid solubility limit; that is, when the amount of impurity atoms exceeds the solid solubility limit, precipitates form at the grain boundaries^35^. These precipitates can then block the movement of grain boundaries with the pinning force of second-phase particles, which is described in the so-called Zener pinning model^36^. In general alloys, both precipitates and solute atoms can suppress grain growth simultaneously through both thermodynamic and kinetic stabilization^37^. Although nanoscale precipitates effectively impede grain growth, they are also thermally unstable and easily coarsen at high temperatures, thus limiting their potential.

As previously mentioned, the effects of solute atoms and precipitates on grain growth suppression are thermodynamically restricted. To overcome this limitation of alloy materials, the thermal stability of nanocomposite materials has been investigated to determine their suitability for high-temperature applications^38–40^. Since reinforcement can suppress the movement of grain boundaries without the formation of secondary phases between the matrix and reinforcement materials, it is expected that nanocomposites can maintain their high strength and elastic moduli, even at elevated temperatures^41–44^. In general, the effect of reinforcement on grain growth suppression was found to vary with morphology. Zero-dimensional (0D) reinforcements, with aspect ratios close to one (e.g., fullerene and ceramic nanoparticles), suppress grain growth through pinning effects similar to those seen for precipitates in alloy materials^45^. Furthermore, unlike precipitates, the reinforcements offer the benefits of controllable sizes and volume fraction for enhancing the Zener pinning force. One-dimensional (1D) reinforcements with high aspect ratios, such as carbon nanotubes (CNTs), have been shown to effectively hinder grain growth in multiple directions due to their ability to be stretched at the grain boundaries^46–49^. The ideal reinforcement materials for suppressing grain growth at high temperatures through kinetic stabilization, however, are two-dimensional (2D) materials, such as graphene, due to the possibility of suppressing atomic diffusion across the grain boundaries as well as pinning grain boundaries^50^.

Although decades of research has mainly focused on composites reinforced with carbon nanomaterials^51,52^, boron nitride (BN) nanomaterials can also be considered as suitable reinforcement materials for structural composites operated at high temperatures, due to not only their thermal stability^53,54^, but also desirable mechanical properties (e.g., elastic modulus of ~1 TPa and tensile strength of ~30 GPa^55^). Furthermore, BN nanomaterials may be more stable in the Al matrix compared with carbon nanomaterials, which easily react with Al to form carbides^56^. Some research has been reported on the fabrication and strengthening effect of BN at room temperature in Al composites reinforced with BN nanomaterials^56–59^. The microstructural/mechanical stability of BN-reinforced Al composites at high temperatures, however, has yet to be studied thoroughly.

In this study, we have investigated the effect of 2D BN nanosheets (BNNS), which are mechanically exfoliated from h-BN, on grain growth suppressing behaviors at high temperatures in Al-based nanocomposites. The microstructure and mechanical properties of the BNNS-reinforced Al composites were investigated after heat treatment at 580 °C (i.e., 0.91 *T*_m_, where *T*_m_ is the melting point of Al) for 1, 2, 3, 6, 12, 24, 72, and 120 h. This work aims to examine the structural effects of BNNS on grain growth suppression at high temperatures as functions of the reinforcement volume, shape, and orientation using a modified Zener pinning model along with phase field simulations.

## Results

### Mechanically exfoliated BNNS reinforced Al nanocomposite

**Figure 1:**
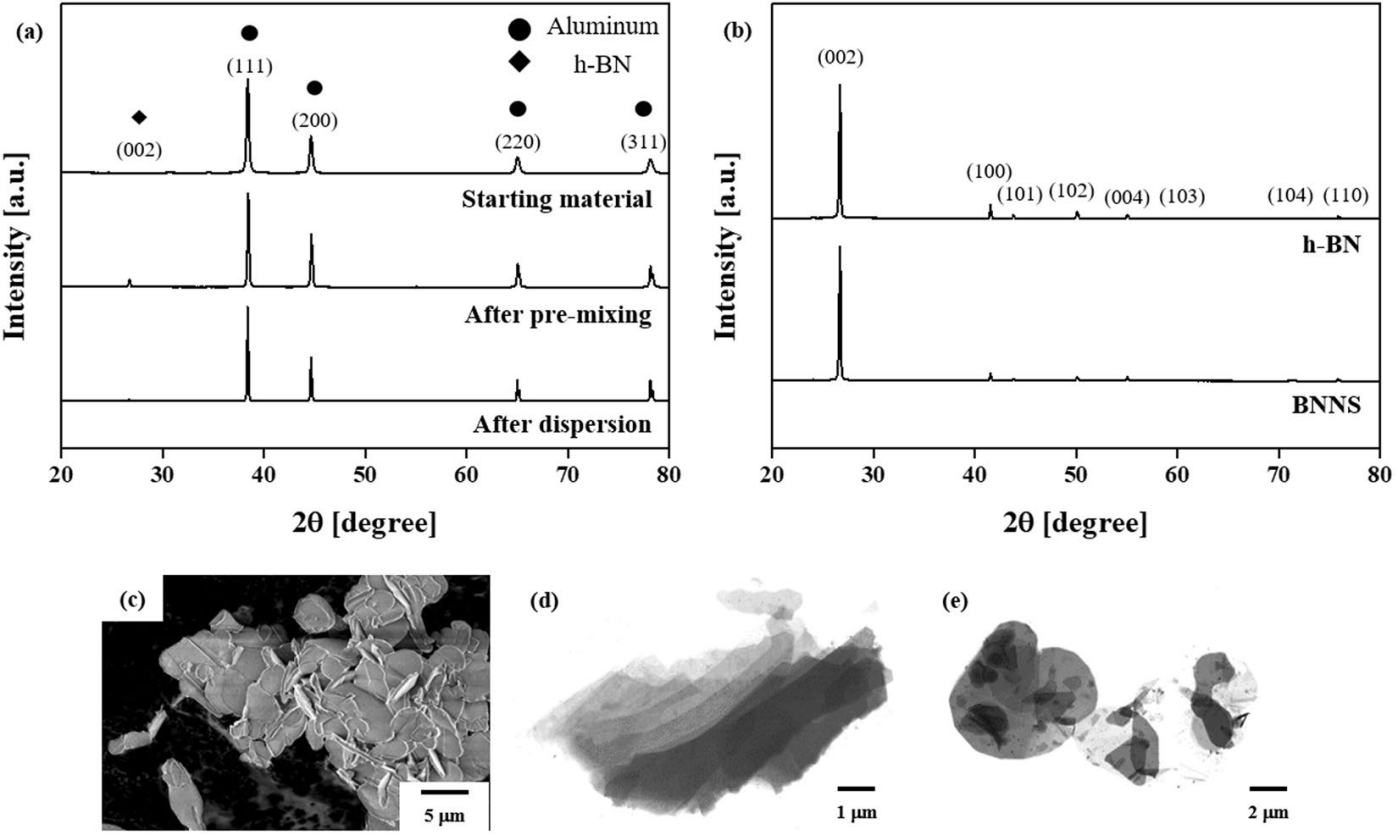
(**a**) XRD patterns of Al and h-BN starting powders and ball-milled powders after pre-mixing and dispersion, and (**b**) comparison between h-BN and exfoliated BNNS, and SEM images of (**c**) h-BN powder and (**d**,**e**) exfoliated BNNS. After wet ball milling, the h-BN is slid by shear force, resulting in exfoliation into few-layer BNNS.

Figure 1(a) shows XRD patterns of simply mixed Al and 2 vol.% h-BN powder (starting materials), pre-mixed Al/BNNS powder, and attrition-milled Al/BNNS composite powder. All powders show peaks corresponding with the (111), (200), (220), and (311) planes of Al, while a notable peak for the (002) plane of h-BN is observed only for the pre-mixed powder. Comparisons of the h-BN and exfoliated BNNS XRD patterns shown in Fig. 1(b) also exhibit similar intensity differences; the intensity (*I*) ratio between the (002) and (100) planes (I_(002)_/I_(100)_) increases from 9.31 in the starting materials to 18.43 in the pre-mixed powders. The significant increase in the peak intensity of the (002) h-BN reflection can be attributed to exfoliation of h-BN into BNNS during wet ball milling, leading to a substantial increase in the preferred orientation^60^. During the wet ball milling from the pristine h-BN powder (Fig. 1(c)), a shear force was induced on h-BN from the contact between the rotating balls and h-BN. As a result, some BN layers could slide, as shown in Fig. 1(d). A planetary mill that only involves rotation of bowls without impellers is beneficial for applying shear forces without significant material fracture^61^. Ethanol was used to weaken the van der Waals forces between the BN layers by strongly interacting with the top layer of h-BN^62^. After wet ball milling, h-BN was shown to be in the form of exfoliated transparent few-layer BNNS (Fig. 1(e)).

### Microstructural observation of Al/BNNS nanocomposites

**Figure 2:**
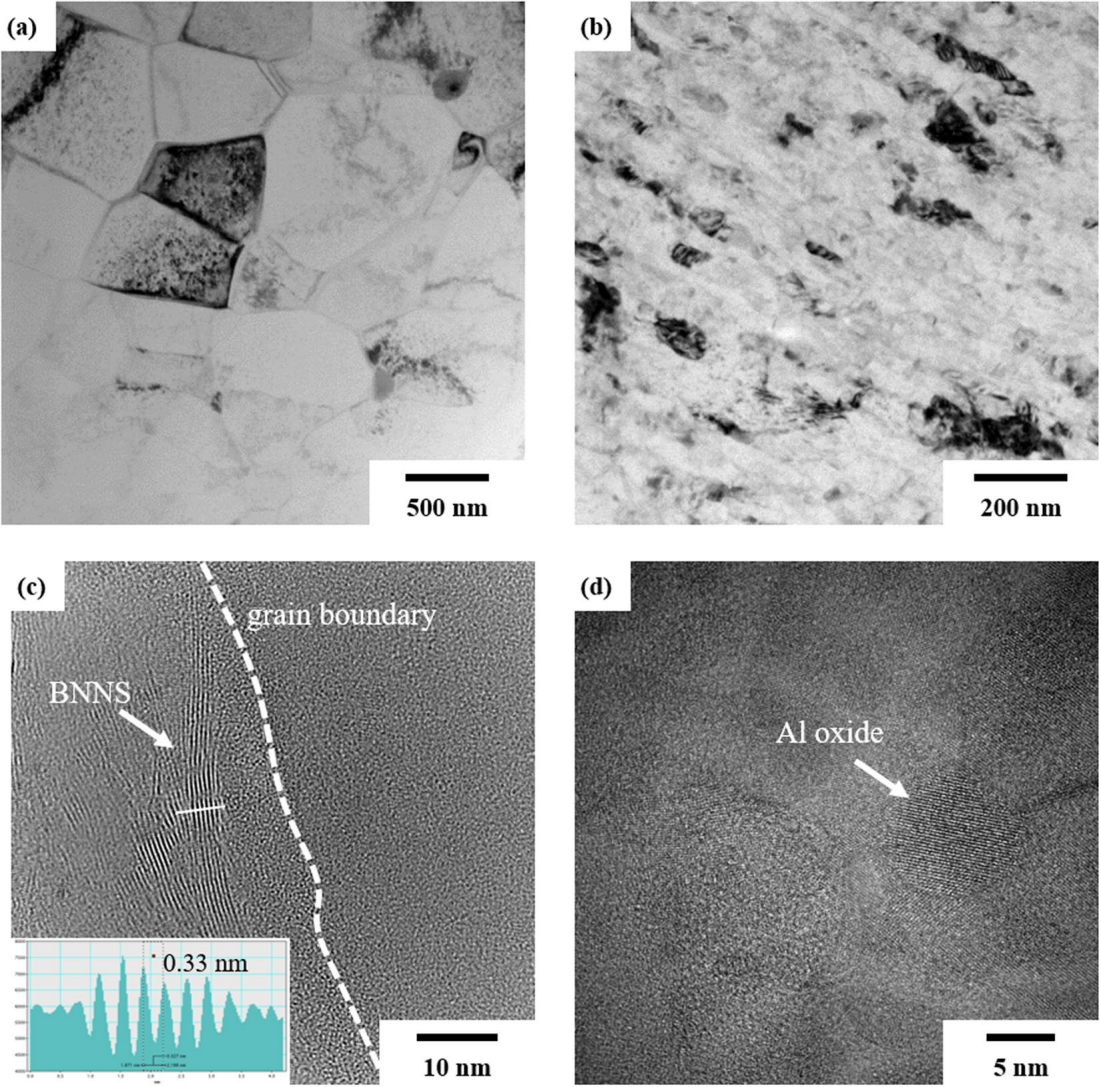
TEM images of (**a**) pure Al and (**b**–**d**) Al/BNNS composites. Al grains are refined by dynamic recrystallization during high-energy ball milling, and BNNS is observed at the grain boundaries.

Figure 2 shows TEM images of (a) pure Al and (b-d) Al/BNNS composites. During high-energy ball milling, Al grains are refined from 600 to 45 nm by dynamic recrystallization that involves an extremely large strain stored from repeated plastic deformation^63^. Figure 2(c) and Supplementary Fig. S1 show few-layer BNNS dispersed in the Al matrix, and the line profile (inset) of the image intensity shows that the d-spacing of the (002) plane of BNNS is close to the value reported by the International Centre for Diffraction Data (ICDD) (d_(002)_ = 0.33 nm, reference code 98-002-4644). Furthermore, it clearly shows a well-bonded and clean interface between Al and BNNS. The interface plays an important role in enhancing nanocomposite performance^64^. The presence of Al oxides, as indicated by the EDS analysis shown in Supplementary Fig. S2, were also evident at the grain boundaries (Fig. 2(d)). Al oxide precipitates were also observed in monolithic Al after heat treatment.

### Grain growth behavior in monolithic Al and Al/BNNS composites

**Figure 3:**
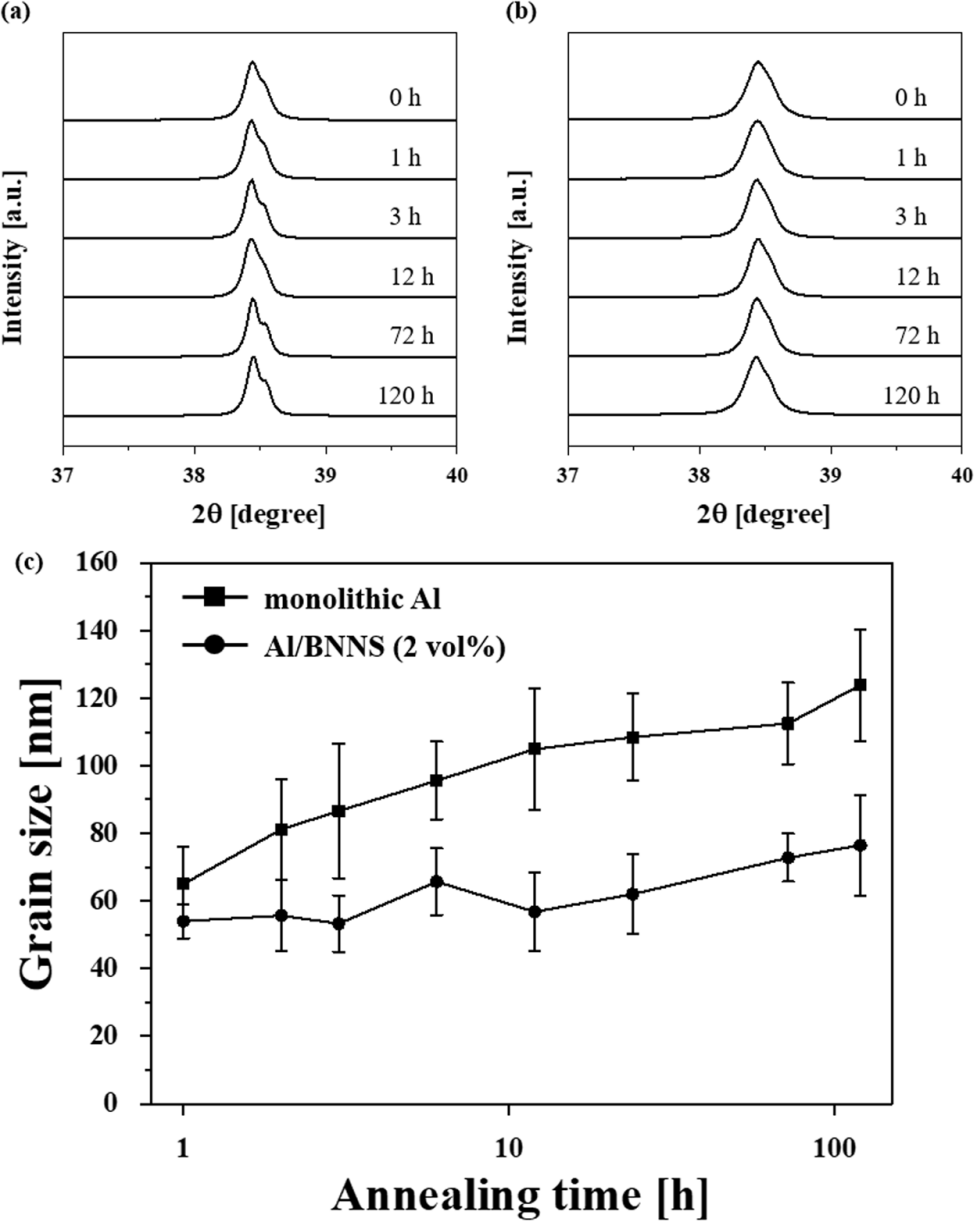
XRD patterns of (**a**) monolithic Al and (**b**) its composites reinforced by 2 vol.% of BNNS after heat treatment at 580 °C for 0, 1, 3, 12, 72, and 120 h, and (**c**) their variation of grain size as a function of heat treatment time. After heat treatment for 120 h, peak separation into K_α1_ and K_α2_ is observed only in monolithic Al; grain growth in the Al/BNNS composite may be suppressed by BNNS.

To investigate the grain growth behavior in monolithic Al and the Al/BNNS composite, heat treatment was conducted at 580 °C for 1, 2, 3, 6, 12, 24, 72, and 120 h. Figure 3 shows XRD patterns with an enlarged (111) reflection from Al of (a) monolithic Al and (b) Al/BNNS composites after heat treatment for 0, 1, 3, 12, 12, 72, and 120 h. After heat treatment for 120 h, monolithic Al shows Kα_1_ and Kα_2_ peak separation, which is not observed in the Al/BNNS composites. A possible explanation for this observation is that the grains in monolithic Al grow significantly during the heat treatment, leading to sharper Kα_1_ and Kα_2_ peaks. In order to investigate the role of BNNS on the suppression of grain growth, the average grain sizes of monolithic Al and Al/BNNS composite were compared as a function of the heat treatment time, as shown in Fig. 3(c). The average grain size was calculated from the XRD data using the Williamson–Hall equation as follows^65^:

3 $$\beta \,\cos \,{\theta }_{B}=\frac{{k}_{B}\lambda }{D}+4\varepsilon \,\sin \,{\theta }_{B}$$

where *β* is full width at half maximum, *k*_*B*_ is a dimensionless constant (0.94), *λ* is the wavelength of Cu K_α_ radiation (0.1541 nm), ${\theta }_{{\rm{B}}}$ is the Bragg angle, *D* is the grain size, and *ε* is the micro strain. The squares and circles in Fig. 3(c) correspond to the grain size of monolithic Al and Al/BNNS composites heat treated for 1, 2, 3, 6, 12, 24, 72, and 120 h, respectively, and the error bar indicates the deviation in grain size. After heat treatment for 120 h, the average grain size of monolithic Al and the Al/BNNS composite increases from 47 to 124 nm and from 39 to 76 nm, respectively. These trends are consistent with the TEM results; monolithic Al undergoes more significant grain growth (120%) than the Al/BNNS composites (83%), as determined by the linear intercept method from the TEM images of monolithic Al and the Al/BNNS composites after heat treatment for varied times, as shown in Supplementary Figs S3 and S4. The greater increase in grain size for monolithic Al clearly indicates that the BNNS dispersed in the Al matrix effectively suppresses the grain growth of Al at high temperatures. By fitting the experimental data (i.e., grain size variation with heat treatment time) using Eq. (2), the grain growth exponent was obtained to be approximately 9 and 11 for monolithic Al and Al/BNNS composite, respectively, as shown in Supplementary Fig. S5. The high values may result from grain boundary pinning by the impurities and Al oxides generated during ball milling, as well as the presence BNNS in the Al composites.

### Variation in yield strength of monolithic Al and Al/BNNS composites

**Figure 4:**
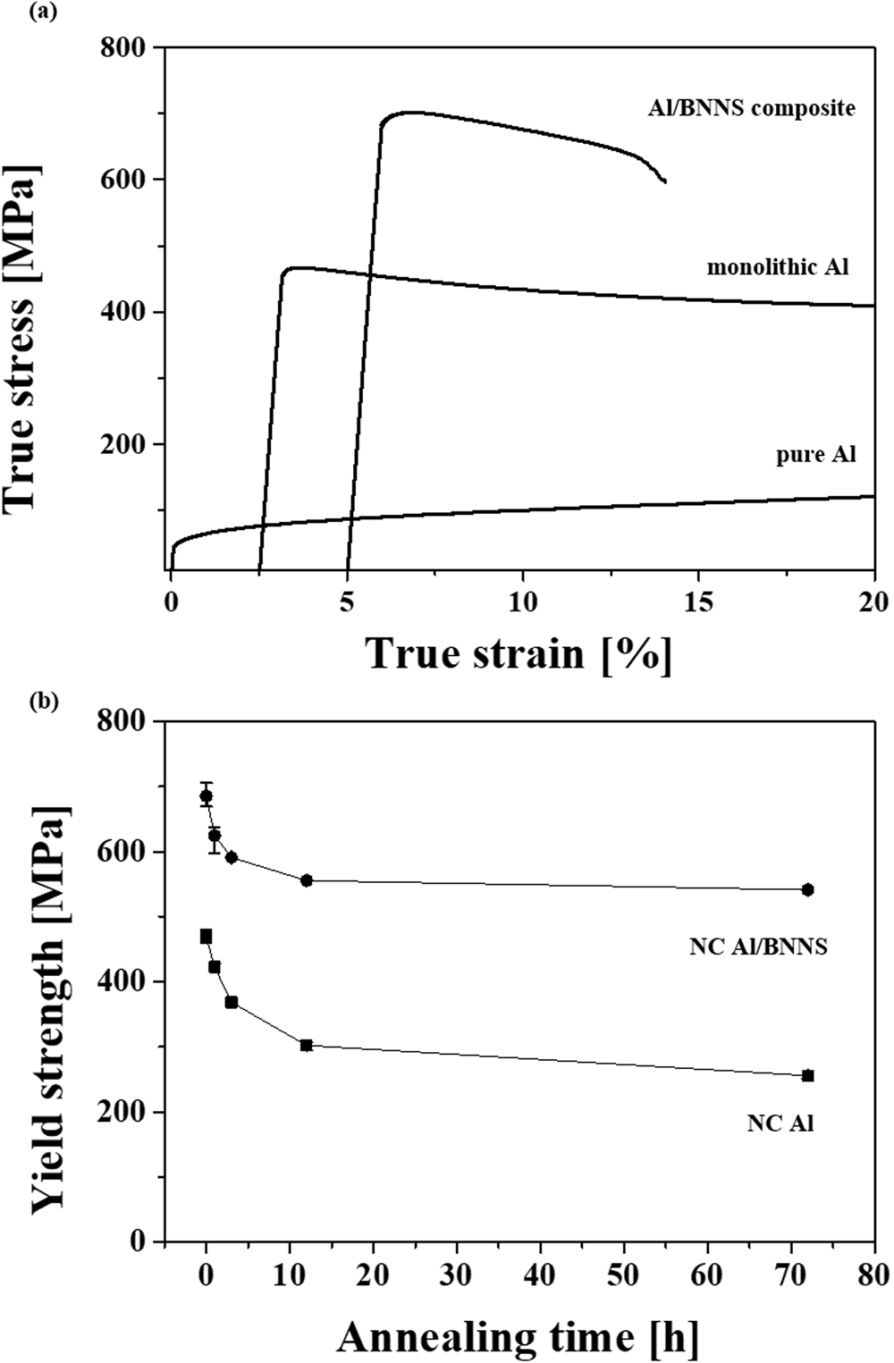
(**a**) Compressive true stress-true strain curves of pure Al, monolithic Al, and Al/BNNS composite, and (**b**) the variation for yield strength of NC Al and NC Al/BNNS as a function of annealing time. Compared to pure Al, the yield strength of monolithic Al significantly increases due to boundary strengthening. The Al/BNNS composite exhibits a yield strength of 685 MPa due to the reinforcing effect. The decreasing rate of the yield strength after heat treatment for 72 h in monolithic Al and the Al/BNNS composite is 45 and 20**%**, respectively.

Figure 4(a) shows compressive true stress–true strain curves of pure Al (no ball milling), monolithic Al, and the Al/BNNS composite. The yield strength of monolithic Al significantly increases to 468 MPa, compared to only 80 MPa for pure Al because of boundary strengthening. Further, the Al/BNNS composite exhibits a yield strength of 685 MPa due to the reinforcing effect of the BNNS. The evolution of the yield strength ($${\sigma }_{y}$$) of monolithic Al and the Al/BNNS composites after heat treatment for 1, 3, 12, and 72 h is shown in Fig. 4(b). Although the strength decreases with increasing heat treatment time, the decreasing rate of the yield strength after heat treatment for 72 h (i.e., $$\frac{{\sigma }_{y,0h}-\,{\sigma }_{y,72h}}{{\sigma }_{y,0h}}\times 100$$$$)$$ in monolithic Al and the Al/BNNS composite is calculated as 45 and 20%, respectively. The mechanical properties of crystalline materials are mainly dependent on the microstructure (e.g., grain size, dislocation density, lattice distortion, and size/shape distribution of precipitates). The primary strengthening mechanism can be examined using boundary strengthening, which is also known as the Hall–Petch relationship^66^:

4 $${\sigma }_{y}={\sigma }_{0}+{k}_{hp}{D}^{-1/2}$$

where ${\sigma }_{y}$ is the yield strength, ${\sigma }_{0}$ is the intrinsic strength, and *k*_*hp*_ is the strengthening coefficient. Thus, the difference in the decreasing rate of the strength between monolithic Al and the Al/BNNS composite may be caused by the grain growth suppression effect of BNNS on the Al matrix. Moreover, Vickers’ hardness tests for the Al/BNNS composites were conducted parallel and normal to the pressing direction in order to investigate the extent of alignment of BNNS, as shown in Supplementary Fig. S6. In this study, the hardness value measured parallel to the pressing direction was similar to that measured normal to the pressing direction (consistent with isotropic properties), indicating that the BNNS was dispersed with random orientation in the Al matrix.

## Discussion

In this study, the microstructural and mechanical behaviors of monolithic Al and Al/BNNS composites have been investigated. After the heat treatment for 72 h, the strength of monolithic Al decreased from 468 to 256 MPa (approximately 45%) because of grain growth, while the decreasing rate of the Al/BNNS composite was only 20%. As boundary strengthening is the major strengthening mechanism for monolithic Al and Al/BNNS composites, strength is mainly varied according to grain size evolution during heat treatment. According to the investigation involving grain size evolution (Fig. 3), the grain sizes of monolithic Al and the Al/BNNS composite increased from 47.3 and 39.4 nm to 104.9 and 56.8 nm after heat treatment for 12 h, respectively, and the grain sizes increased further to 112.5 and 72.8 nm after 72 h. These results demonstrate that the grain growth rate (*dD*_*t*_/*d*_*t*_) decreased with increasing heat treatment time; the corresponding rates for monolithic Al and the Al/BNNS composite are 4.8 and 1.45 nm/h for 12 h, whereas those for the range between 12 and 72 h are reduced to 0.13 and 0.27 nm/h. Owing to the high driving force for grain growth, it initially occurred very rapidly. The rate then gradually decreased as the heating time increased, resulting in changes in softening behavior, as shown in Fig. 4(b); indeed, the strength became more rapidly reduced at relatively short durations (i.e., <12 h) than after 12 h. However, the decreasing rate of the strength in the Al/BNNS composite is significantly lower than that of monolithic Al, which may result from the suppression of grain growth caused by the reinforcement in the Al/BNNS composite.

**Figure 5:**
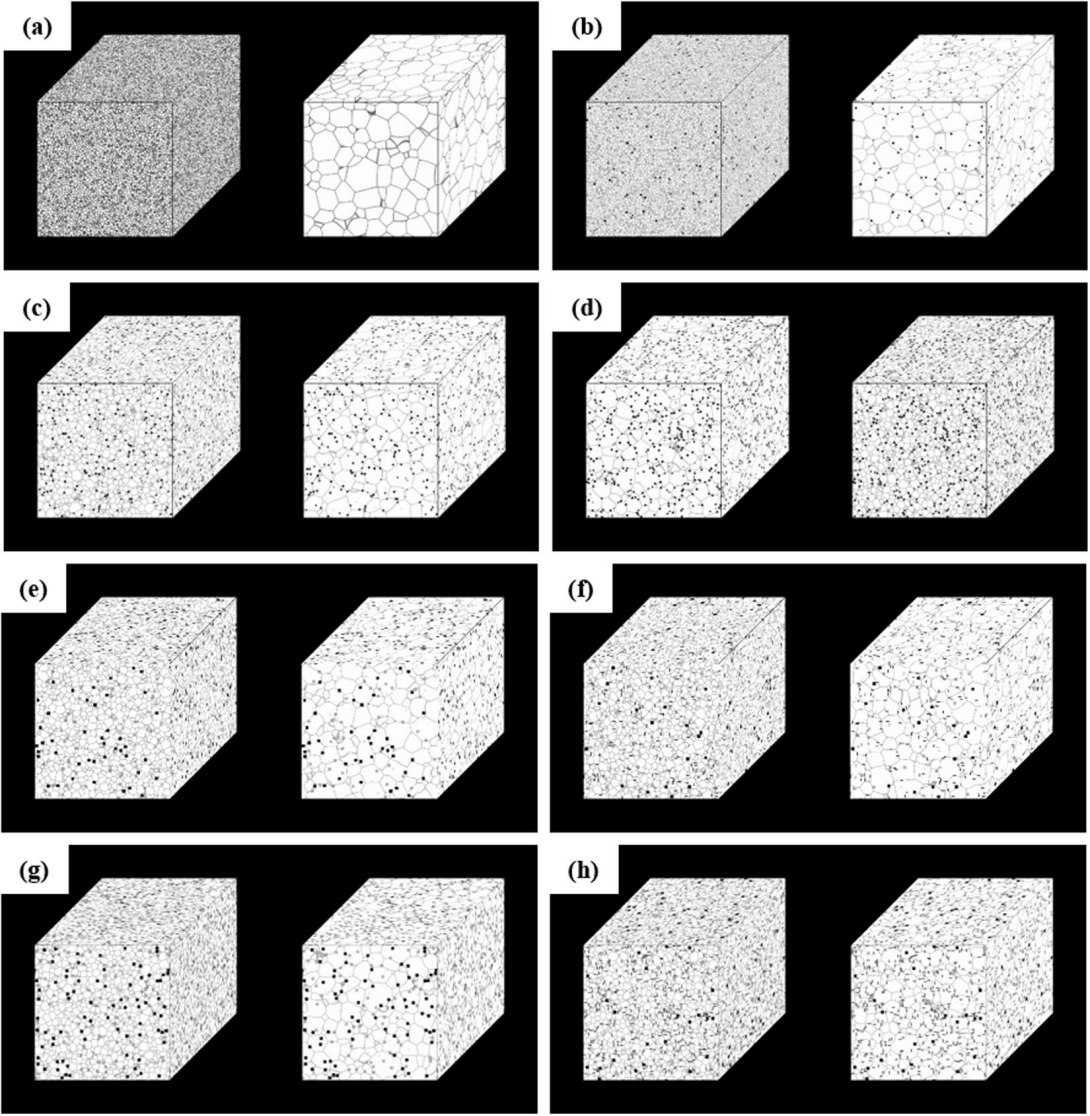
3D microstructures generated via phase-field grain-growth simulations at initial state and final states of (**a**) 0, (b) 1, (**c**) 3, and (**d**) 5 vol.% spherical particle-containing composites and 3 and 5 vol.% plate-like particle-containing composites assuming (**e** and **g**) unidirectional alignment and (f and h) random orientation.

**Figure 6:**
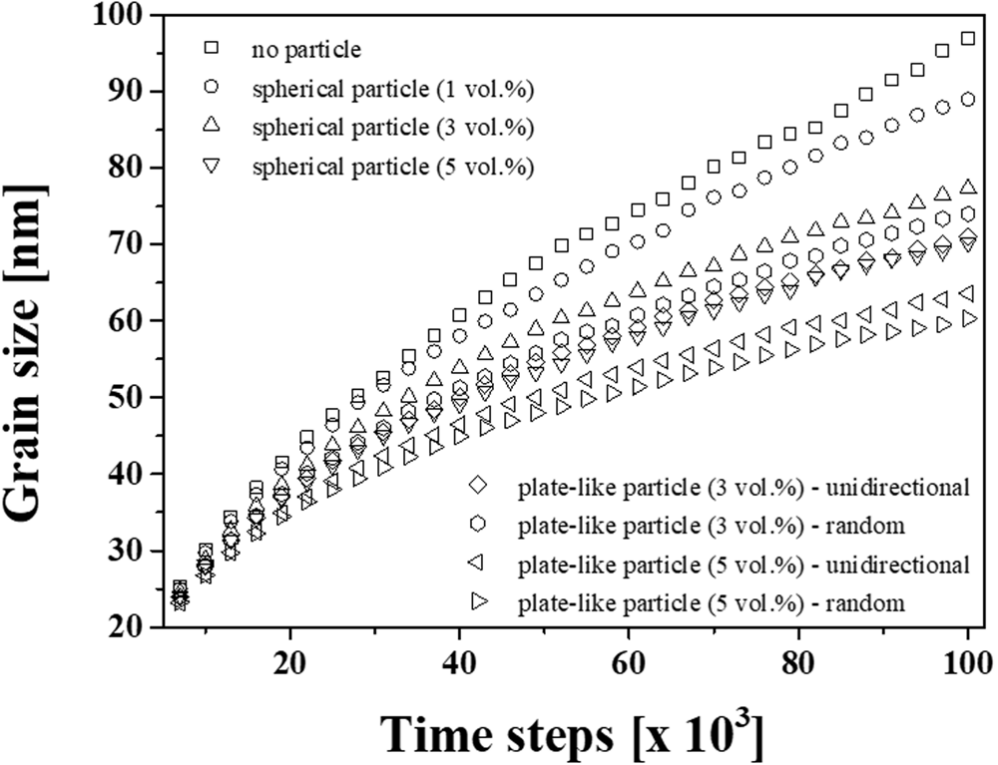
Variation of the average grain size for spherical and plate-like particle-containing composites as a function of time. The saturated grain size decreases with increasing volume fraction of the particles. The grain boundary movement is most effectively suppressed by the randomly oriented plate-like particles.

For composite materials, the fundamental reinforcement microstructural features such as volume fraction, size, shape, orientation, as well as the degree of distribution of reinforcement have been considered to act as suppression factors for grain boundary pinning at high temperatures. The Zener pinning effect has been previously studied using computer simulations in order to investigate the microstructural effect on grain size evolution^67^. To qualitatively analyze the effect of secondary phase materials (i.e., BNNS and Al oxide particles) on grain growth suppression, phase-field simulations were performed involving heat treatment of particle-containing Al. Based on microstructural observation, BNNS and oxide particles were assumed to be plate-like particles with sizes of 15 (width, *w*_s_) × 15 (length, *l*_s_) × 2 (thickness, *t*_s_) nm^3^ and spherical particles with diameters of 15 nm, respectively. Additionally, the simulation was conducted with 1, 3, and 5 vol.% spherical particles as well as 3 and 5 vol.% plate-like particles with unidirectional and random orientation to study the effect of orientation, shape, and volume fraction of particles. To this end, Fig. 5 shows the 3D microstructures generated via phase-field grain-growth simulations at the initial and final states of the composites containing spherical particles with various volume fractions (i.e., (a) 0, (b) 1, (c) 3, and (d) 5 vol.%) and 3 and 5 vol.% plate-like particles assumed to be unidirectionally aligned (e and g) or randomly oriented (f and h). The variations in the average grain size for composites containing the spherical and plate-like particles as a function of the simulation time is summarized in Fig. 6. These results show that the saturated grain size during heat treatment (i.e., the Zener limit, *D*_z_) decreases with increasing volume fraction of the particles. Furthermore, while the size of plate-like particles is smaller than that of spherical particle despite the same volume fraction, the randomly oriented plate-like particles exhibit the lowest value.

**Figure 7:**
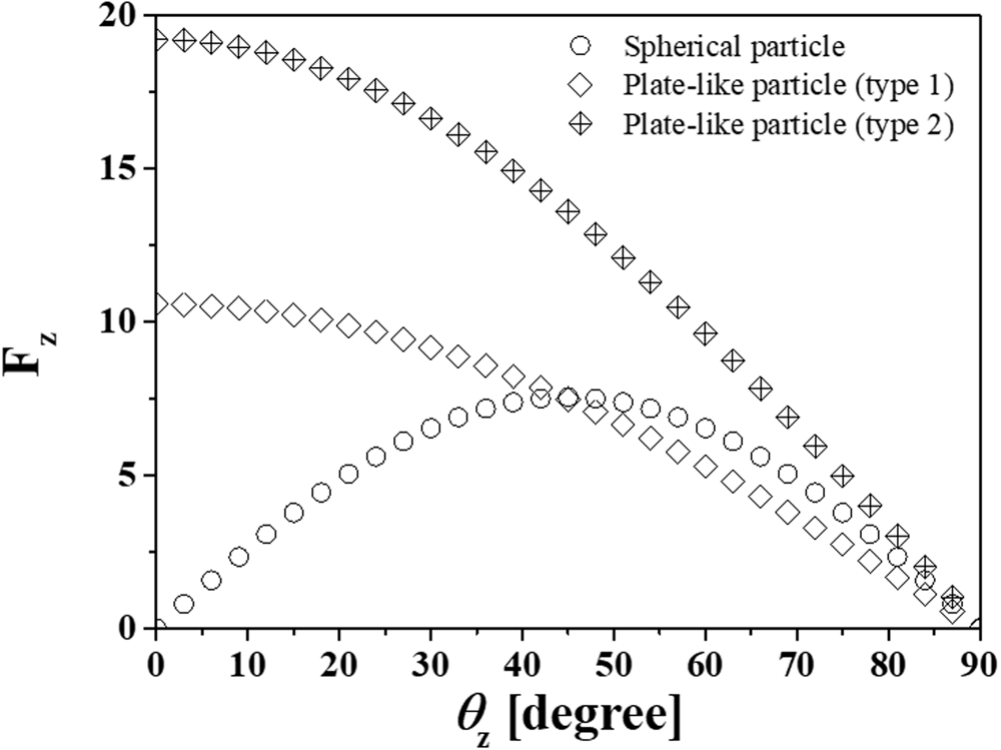
Variation of the Zener pinning force of spherical and plate-like particles as a function of *θ*_Ζ_. The pinning forces for the plate-like particles are calculated with consideration of the aligned direction of BNNS; types 1 and 2 represent parallel and normal movements of the grain boundary to the alignment direction of BNNS, respectively. When the maximum and total pinning force of the spherical and oriented plate-like particles is compared as a function of the angle, the pinning effect of the plate-like particles is more significant than spherical particles, regardless of their orientation. The plate-like particles with parallel orientation to the boundary movement direction give rise to the most significant pinning effect.

The efficiency for grain growth suppression of the secondary phase particles can be assumed to be a relative comparison of increasing grain size over the same time period with a particle-free composite ($i.e.,\,\frac{{\rm{\Delta }}{D}_{t}^{no-particle}-\,{\rm{\Delta }}{D}_{t}^{composite}}{{\rm{\Delta }}{D}_{t}^{no-particle}}\times 100$, where Δ*D*_*t*_ = *D*_*t*_ − *D*_0_. When the grain size of the particle-free composite (0 vol.%) increases as 60 nm, spherical particles give an efficiency of 7.3, 18.7, and 28.1% for volume fractions of 1, 3, and 5 vol.%, respectively, and the efficiency value for the randomly and unidirectional oriented 3 vol.% plate-like particles is 26.4 and 23.8%, respectively. Moreover, 5 vol.% plate-like particle-containing composite reveals the efficiencies of 40.2 and 35.7% depending on the random and unidirectional orientation. As mentioned before, the volume fraction, shape, and orientation of secondary phase particles influence the suppression of grain growth in composite materials. The volume fraction (*f*) effect of secondary phase materials with radius (*r*) on the Zener limit can be expressed as follows^68^:

5 $${D}_{Z}=\frac{{k}_{z}r}{{f}^{m}}=\frac{{k^{\prime} }_{z}}{{S}_{v}{f}^{m}}$$

where *k*_*z*_ is a dimensionless constant, *S*_*v*_ is the surface area per unit volume of the particle, and *m* is an index for the volume fraction. This shows that the grain size has an inverse relationship with the concentration of secondary phase materials. The simulated grain size also decreases with increasing spherical phase concentration. Furthermore, while the suppression effect of the spherical particles on grain growth was found to be less than that of the plate-like particles regardless of orientation due to high specific surface area, the randomly oriented plate-like particles were the most effective for suppressing grain boundary movement at high temperatures. To discuss the shape effect on grain growth suppression, the pinning force of an individual plate-like particle was calculated and compared to that of a spherical particle. The Zener pinning force per spherical particle (${F}_{z}^{{sphere}}$) can be expressed using the following equation^69^:

6 $${F}_{z}^{sphere}=(2\pi rsin({\theta }_{z}))\cdot ({\gamma }_{GB}\,\cos ({\theta }_{z}))$$

where *r* is the particle radius, and ${\theta }_{z}$ is the angle between the direction of the boundary movement and the pinned boundary. The equation for the Zener pinning force is separated into two terms: (1) total contact line and (2) component of the boundary energy in the direction of the boundary movement. To study the shape effect of secondary phases on boundary pinning, the model was modified for plate-like particles to consider particle alignment; the modified equations are expressed as follows:

7 $${F}_{z}^{plate1}=(2({w}_{s}+{t}_{s}))\cdot ({\gamma }_{GB}\,\cos ({\theta }_{z}))$$

8 $${F}_{z}^{plate2}=(2({w}_{s}+{l}_{s}))\cdot ({\gamma }_{GB}cos({\theta }_{z}))$$

where ${F}_{z}^{plate1}$ and ${F}_{z}^{plate2}$ are the pinning forces of plate-like particles aligned parallel (type 1) and normal (type 2) to the direction of boundary movement, respectively, as shown in Supplementary Fig. S7. It was assumed that the interface energy between the matrix and the secondary particle is isotropic (${\gamma }_{{GB}}$ = 0.24 a/u) and the grain boundary is pinned at the edge of the plate-like particles in contact with it^70^. Figure 7 shows the calculated Zener pinning force for spherical and plate-like particles as a function of ${\theta }_{z}$. Plate-like particles give rise to higher maximum and total pinning forces than spherical particles regardless of their orientation, and the pinning force is more significant when the plate-like particles are oriented normal to the direction of the grain boundary movement. Hence, the efficiency for randomly oriented plate-like particles was higher than that of unidirectionally oriented particles. While the pinning force in randomly oriented plate-like particle-containing composites can induce a grain boundary in all directions, the grain in the composite with unidirectional orientation grows long in just one direction, as shown in Supplementary Fig. S8. Furthermore, the Zener model for spherical particles has been modified to describe the relationship between the Zener limit and the volume fraction of plate-like particles. When the Zener limit has an inverse relationship with a maximum pinning pressure (i.e., ${D}_{z}\,\propto \frac{{\gamma }_{GB}}{{P}_{z}}$, where *P*_*z*_ is the maximum pinning pressure), the pressure for plate-like particles can be described as follows:

9 $${P}_{z}^{Plate}={F}_{max}^{Plate}\cdot {n}_{s}^{Plate}$$

where ${F}_{max}^{Plate}$ is the maximum pinning force per particle and ${n}_{s}^{Plate}$ is their surface density, which may be assumed as:

10 $${n}_{s}^{Plate\,(type\,1)}=\frac{f({w}_{s}+{t}_{s})}{{w}_{s}\cdot {l}_{s}\cdot {t}_{s}}$$

11 $${n}_{s}^{(Plate(type2)}=\frac{f({w}_{s}+{l}_{s})}{{w}_{s}\cdot {l}_{s}\cdot {t}_{s}}$$

Thus, the Zener limit for plate-like particles with consideration of their orientation can be summarized as follows:

12 $${D}_{z}^{Plate\,(type\,1)}\approx \frac{{w}_{s}\cdot {l}_{s}\cdot {t}_{s}}{{({w}_{s}+{t}_{s})}^{2}}\frac{{k^{\prime\prime} }_{z}}{{f}^{m}}$$

13 $${D}_{z}^{Plate\,(type\,2)}\approx \frac{{w}_{s}\cdot {l}_{s}\cdot {t}_{s}}{{({w}_{s}+{l}_{s})}^{2}}\frac{{k^{\prime\prime} }_{z}}{{f}^{m}}$$

**Figure 8:**
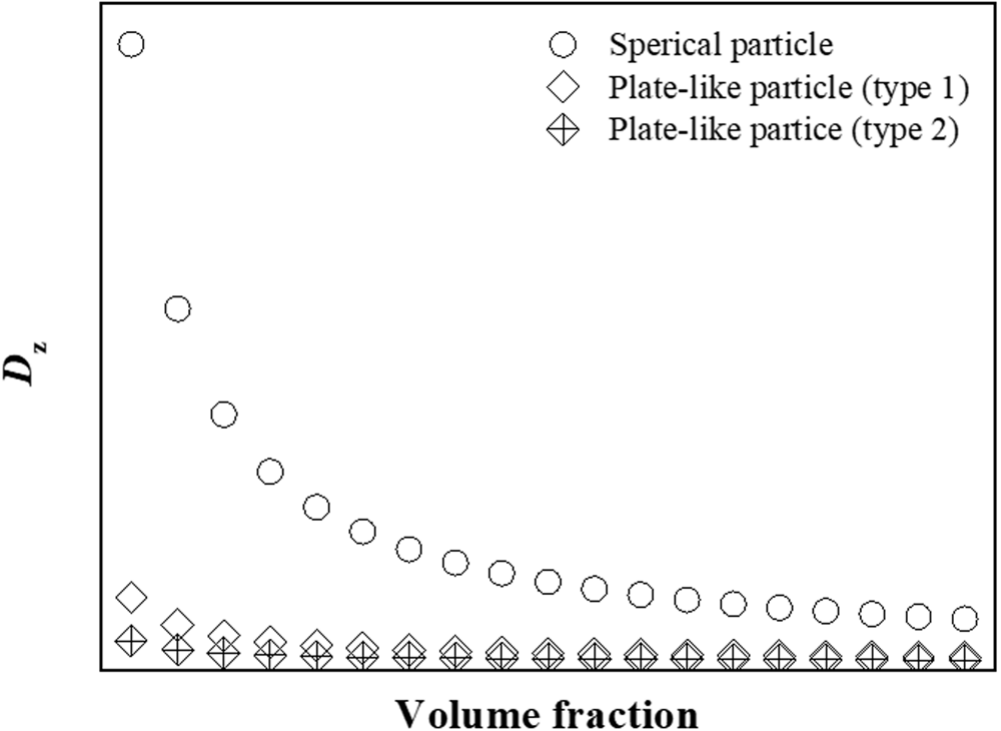
Variation of the Zener limit for spherical and plate-like particles as a function of volume fraction. For the results of the plate-like particles, the Zener limit is calculated with consideration of the aligned direction of BNNS (i.e., parallel (type 1) and normal (type 2)).

Figure 8 shows the variation of the Zener limit for spherical and plate-like particles with consideration of orientation as a function of their volume fraction. Clearly, plate-like particles are more effective for grain growth suppression than spherical particles because of their high pinning force and high specific surface density. Experimentally, the Al/BNNS composite has an efficiency for grain growth suppression of 54.9%; the grain size of the Al/BNNS composite increases from approximately 40 to 67 nm, whereas the size of monolithic Al increase from 47 to 107 nm, according to the fitting results (Supplementary Fig. S5). This may be caused by the suppression effect of randomly oriented BNNS and supported by additionally formed oxide particles. Therefore, 2D plate-like particles (i.e., BNNS) can be considered as effective reinforcements for composite materials with high strength at high temperatures because of the high pinning strength at grain boundaries as well as large specific surface area per volume.

## Conclusion

In this study, we investigated the grain growth suppression effects of BNNS in Al matrix nanocomposites produced via mechanical alloying, followed by hot pressing. High-energy ball milling induced a nanocrystalline grain refinement of Al and dispersed the BNNS, which was exfoliated from h-BN by wet ball milling. Based on the microstructural and mechanical evolution after heat treatment of monolithic Al and the Al/BNNS composite, we can conclude that BNNS effectively suppresses grain growth of the Al matrix and prevents a strength drop. Phase-field simulations were used to qualitatively analyze the effect of secondary phase materials on suppressing grain growth. The simulation results showed that the saturated grain size decreased with increasing particle concentration and that the grain growth was most effectively suppressed by randomly oriented plate-like particles. These results were further supported by calculations of the maximum pinning force. Therefore, 2D structural BNNS can be considered as effective reinforcement for aluminum-based composites, providing high thermal stability.

## Methods

Al-based nanocomposites reinforced with 2 vol% BNNS were produced using a combination of ball milling and hot pressing. First, a three-step ball-milling procedure was used to fabricate the Al/BNNS nanocomposite powder: (1) exfoliation of BN; (2) pre-mixing of BNNS and Al powder; and (3) dispersion of BNNS into the Al powder. Hexagonal BN (h-BN, Kojundo Chemical Laboratory Co. Ltd.) was exfoliated into BNNS using a planetary ball mill (Fritsch, Pulverisette 5, Germany). The h-BN powder was mixed with ethanol (100 mL, 99.5% purity,) for 10 min in a sonicator in order to weaken the van der Waals forces in h-BN. A stainless steel chamber (500 mL) was filled with the h-BN solution and stainless steel balls (5 mm in diameter) with a ball-to-powder weight ratio of 10:1. Then, a 150 rpm milling cycle consisting of 20 min of milling followed by a 40 min pause was repeated 24 times. After vaporizing the ethanol, Al powder (99.5% in purity, Changsung Co., Ltd.) was added to the chamber and ball milling was conducted in order to pre-mix the exfoliated BNNS with the Al powder. The pre-mixing was performed for 12 h and consisted of 8 cycles of 15 min of milling at 200 rpm followed by a 75 min pause. To disperse the exfoliated BNNS into the Al powder, high-energy ball milling was carried out using an attrition mill (KMC-1BV, KMC Co. Ltd.). The mixed Al/BNNS powder was placed into a stainless-steel chamber (containing stainless-steel balls) along with 1 wt.% stearic acid (CH_3_(CH_2_)_16_CO_2_H, Sigma Aldrich Korea Co., Ltd.) as a process control agent with a ball-to-powder ratio of 15:1. Cooling water was circulated around the walls of the chamber to prevent an internal temperature increase during ball milling, which was conducted at 500 rpm for 24 h in an Ar atmosphere. For comparison, the monolithic Al powder was prepared under the same conditions. Then, the ball-milled powders were consolidated into fully compact samples through hot pressing. The powder was placed in a stainless-steel mold with a 30 mm diameter cylindrical cavity. After cold-pressing the powder at room temperature under 30 MPa of pressure, the powder was hot pressed at 500 °C for 1 h under 210 MPa of pressure. To compare the microstructural evolution and mechanical behaviors of monolithic Al and the Al/BNNS composites under different conditions, heat treatment was carried out at 580 °C at a heating rate of 10 °C/min for 1, 2, 3, 6, 12, 24, 72, or 120 h in air using a box furnace (Lenton ECF 12/6).

Phase identification and grain size calculations were carried out using X-Ray diffraction (XRD, Rigaku SmartLab) with Cu K_α_ radiation. The patterns were analyzed using the integrated X-Ray Powder Diffraction Software Package PDXL (Rigaku). Field-emission scanning electron microscopy (FESEM, FEI Verios 460 L) was used to observe the morphology of the exfoliated BN. Microstructural evolution before and after heat treatment was observed using high-resolution transmission electron microscopy (HRTEM, FEI Tecnai G2 F20 460 L). HRTEM samples were prepared using a focused ion beam (FIB, FEI Helios NanoLab 650). Precipitates formed in the Al matrix after heat treatment were observed using scanning transmission electron microscopy-electron energy loss spectroscopy (STEM-EELS, FEI Titan G2) equipped with an energy-dispersive spectrometer (EDS). The degree of BNNS alignment was studied using Vickers hardness tests (Mitutoyo HM-200) conducted with an applied load of 100 g either parallel or normal to the pressing direction. Compression tests were conducted at a strain rate of 10^−3^ s^−1^ using a universal testing machine (UTM, UNITECH-M (RB 301)). Samples with dimensions of 2 × 2 × 3 mm^3^ were prepared by cutting and polishing pressed samples, which were then placed between two tungsten carbide plates coated with a BN lubricant to minimize friction during the tests.

The multi-order parameter phase-field grain growth model^71^, as implemented using the Active Parameter Tracking algorithm^72^, was used in this work. Details of the numerical calculation are provided by Chang *et al*.^67^. In this study, the cell size was 720^3^ and the initial number of grains was 145,000 for more rigor.

## Electronic supplementary material

Supplementary Information
