# Supplementary material for: Structural effect of two-dimensional BNNS on grain growth suppressing behaviors in Al-matrix nanocomposites
Source: Sci Rep. 2018 Jan 25;8:1614. doi: 10.1038/s41598-018-20150-5 (PMC5785491; doi:10.1038/s41598-018-20150-5)
Supplement: Supplementary file 1 — Supplementary Information [file 41598_2018_20150_MOESM1_ESM.pdf]

Structural effect of two-dimensional BNNS on grain growth suppressing behaviors in Al-matrix nanocomposites

Seungjin Nam[1], Kunok Chang[2], Woonki Lee[3], Moon J. Kim[4], Jun Yeon Hwang[3*], Hyunjoo Choi[1]**

[1]School of Advanced Materials Engineering, Kookmin University, Seoul 02707, Republic of Korea

[2]Nuclear Materials Safety Research Division, Korea Atomic Energy Research Institute, Daejeon 34057, Republic of Korea

[3]Institute of Advanced Composite Materials, Korea Institute of Science and Technology, Jeonbuk, 55324, Republic of Korea

[4]Department of Materials Science and Engineering, The University of Texas at Dallas, Richardson, TX 75080, USA

***Corresponding author (e-mail : junyeon.hwang@kist.re.kr)**

****Corresponding author (e-mail : hyunjoo@kookmin.ac.kr)**

Supplementary Table. S1 Comparison of density (ρ), melting temperature (T_m), and activation energy for grain growth by grain boundary self diffusion (Q_B) and lattice diffusion (Q_V) in various metallic materials with crystal structure of FCC, BCC, and HCP

**Material (FCC structure)**	**Ni**	**Cu**	**Ag**	**Al**	**Pb**
ρ [g/cc]	8.9	8.3	10.5	2.7	11.3
T_m [K]	1726	1356	1234	933	601
Q_B [kJ/mol]	115	104	90	84	66
Q_v [kJ/mol]	284	197	185	142	109
**Material (BCC structure)**	**W**	**V**	**Cr**	**Nb**	**Mo**
ρ [g/cc]	19.2	6.1	7.1	8	10.2
T_m [K]	3683	2173	2163	2741	2883
Q_B [kJ/mol]	385	209	192	263	263
Q_v [kJ/mol]	585	308	306	401	405
**Material (HCC structure)**	**Zn**	**Cd**	**Mg**	**α−Ti**	**β−Ti**
ρ [g/cc]	7.12	8.64	1.7	4.54	4.54
T_m [K]	693	594	924	1933	1933
Q_B [kJ/mol]	60.5	54.4	92	97	153
Q_v [kJ/mol]	91.7	76.2	135	150	153

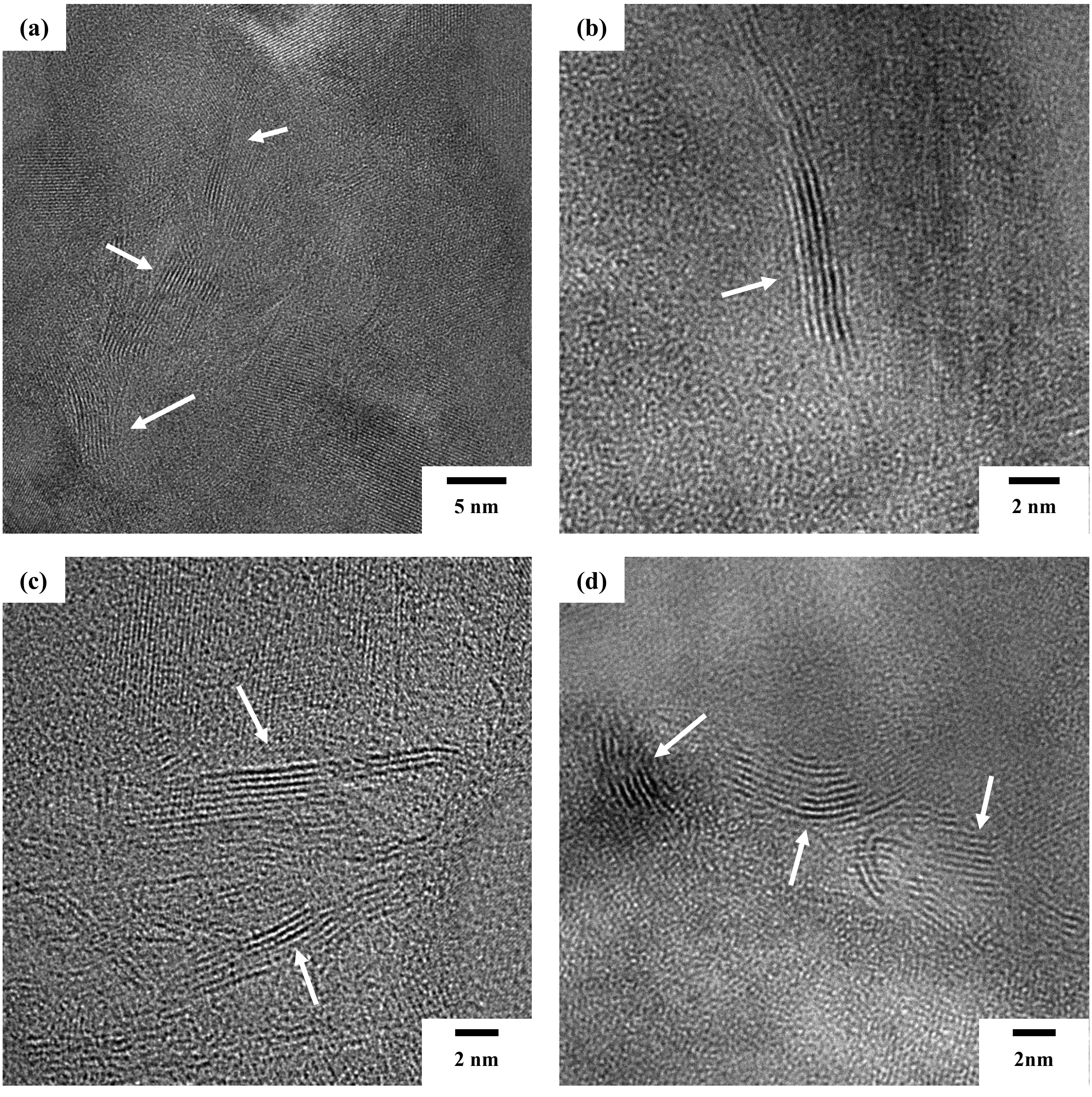

Supplementary Fig. S1 TEM images of BNNS (white arrow) reinforced Al composites.

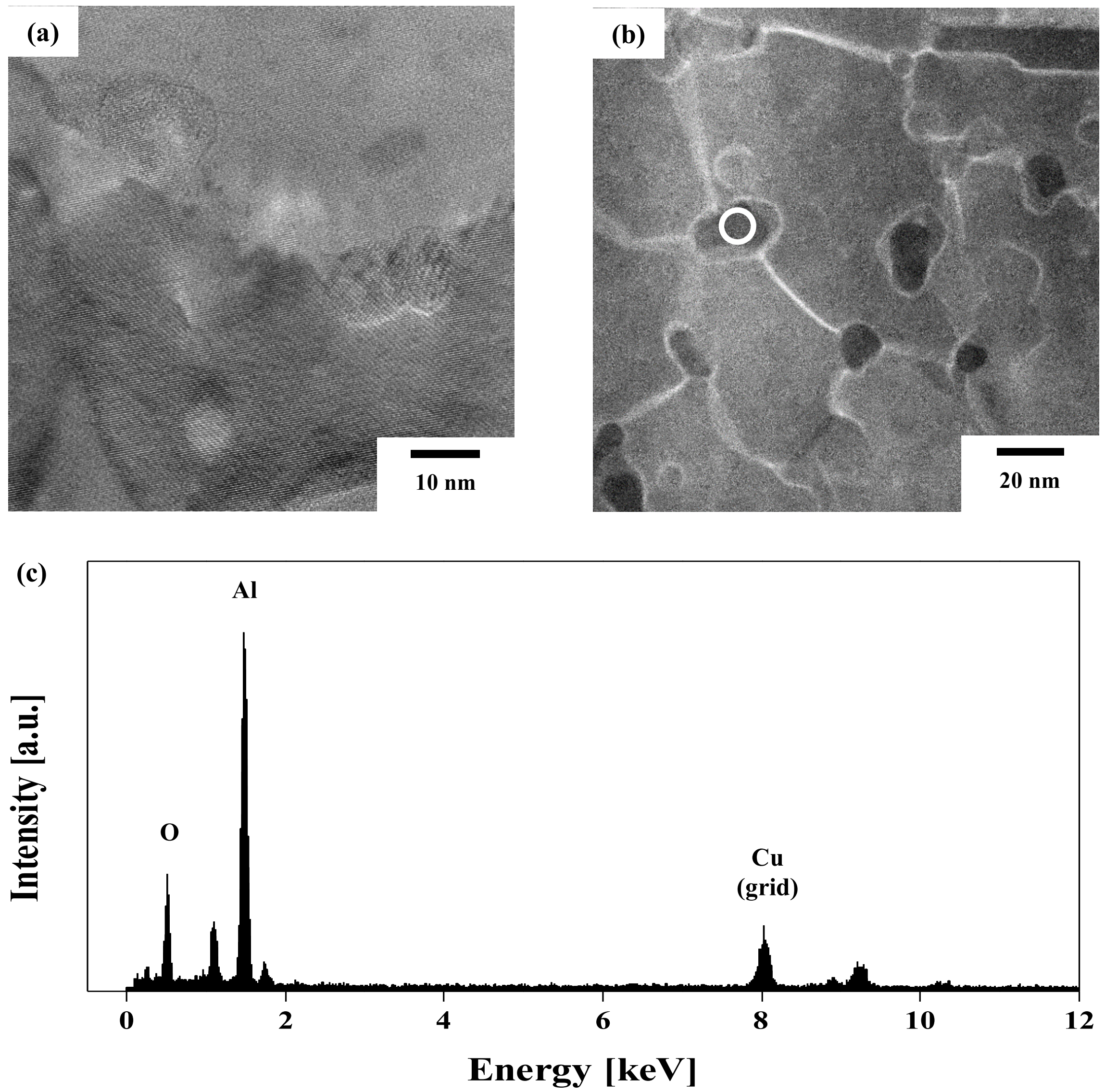

Supplementary Fig. S2 (a) TEM and (b) STEM-HAADF images of Al/BNNS composite after heat-treatment at 580 ℃for 72 h. Supplementary Fig. 2 (c) shows EDS spectra, which is taken from the position marked with a white circle in (b).

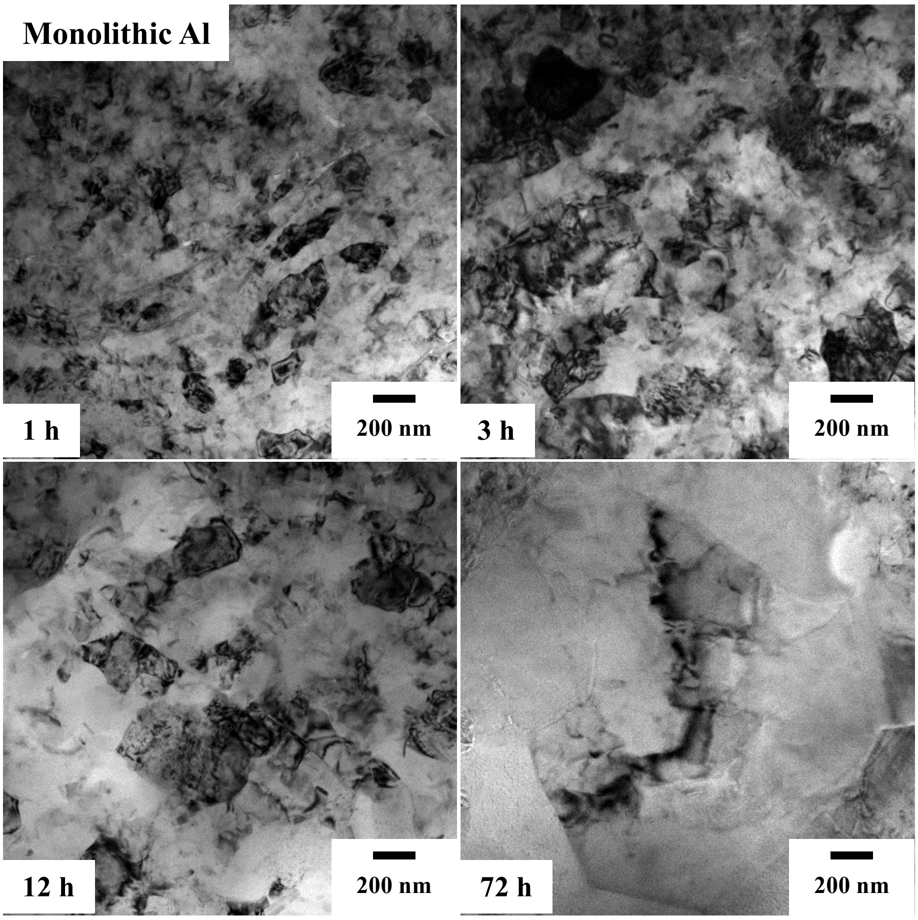

Supplementary Fig. S3 TEM images of monolithic Al after heat-treatment for 1, 3, 12, and 72 h.

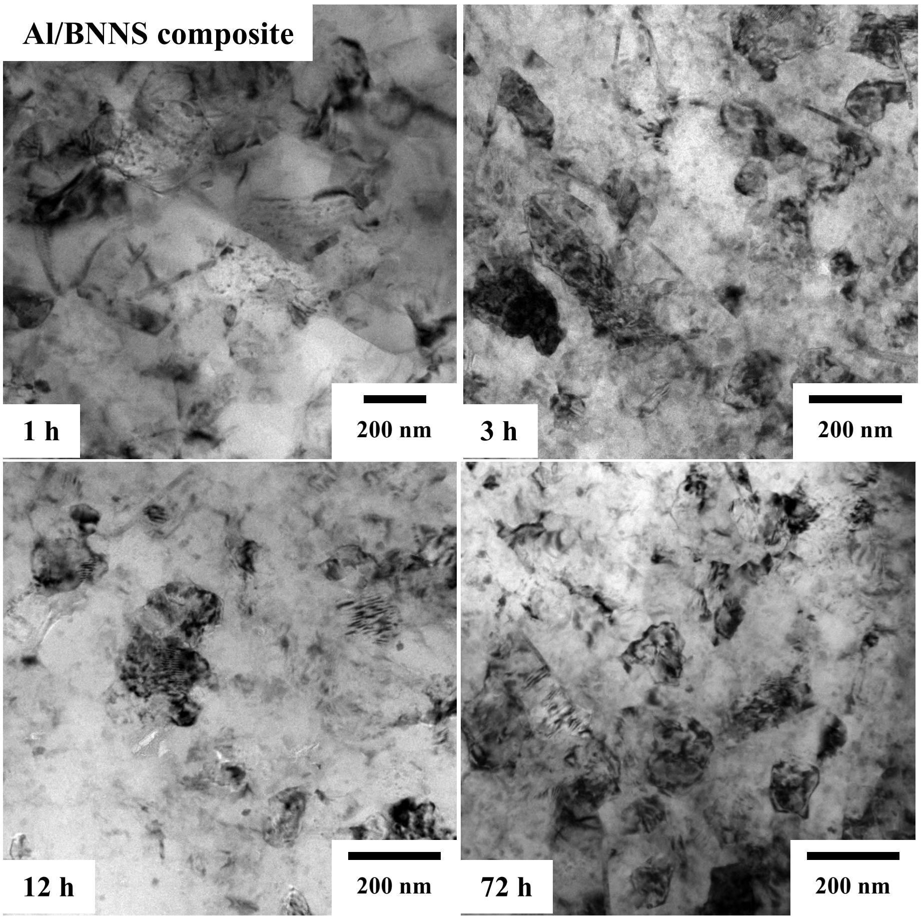

Supplementary Fig. S4 TEM images of monolithic Al after heat-treatment for 1, 3, 12, and 72 h.

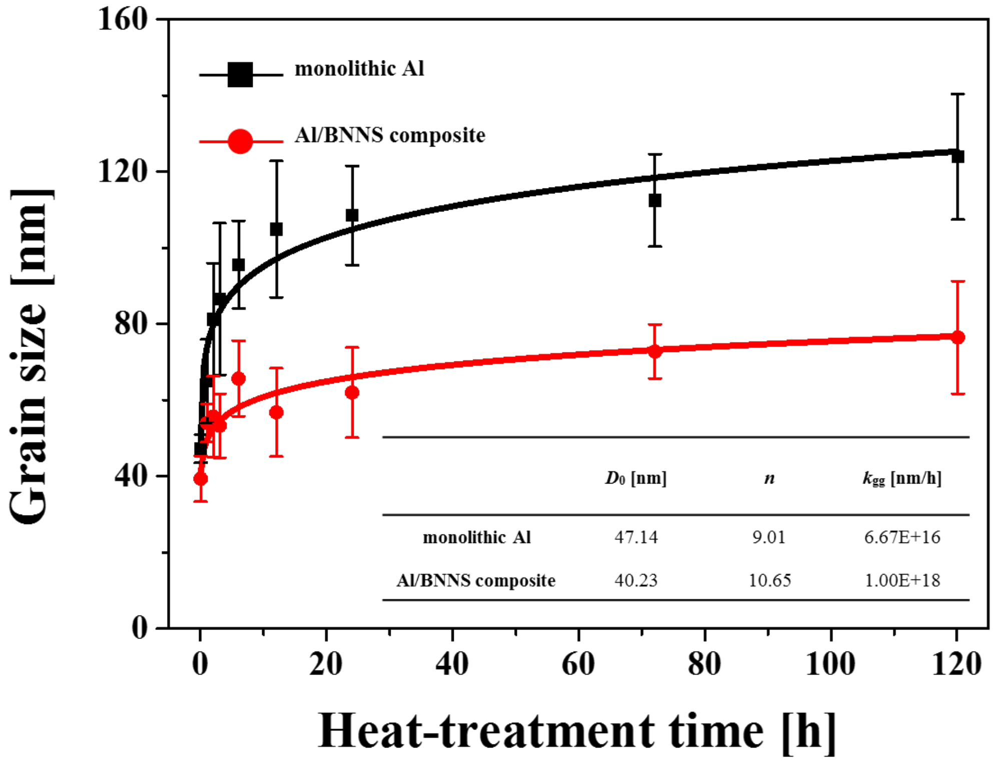

Supplementary Fig. S5 Grain size measured from XRD results and grain size evolution calculated by Eq. 2 with the calculated values in inserted table for monolithic Al and Al/BNNS composite after heat-treatment for varied times.

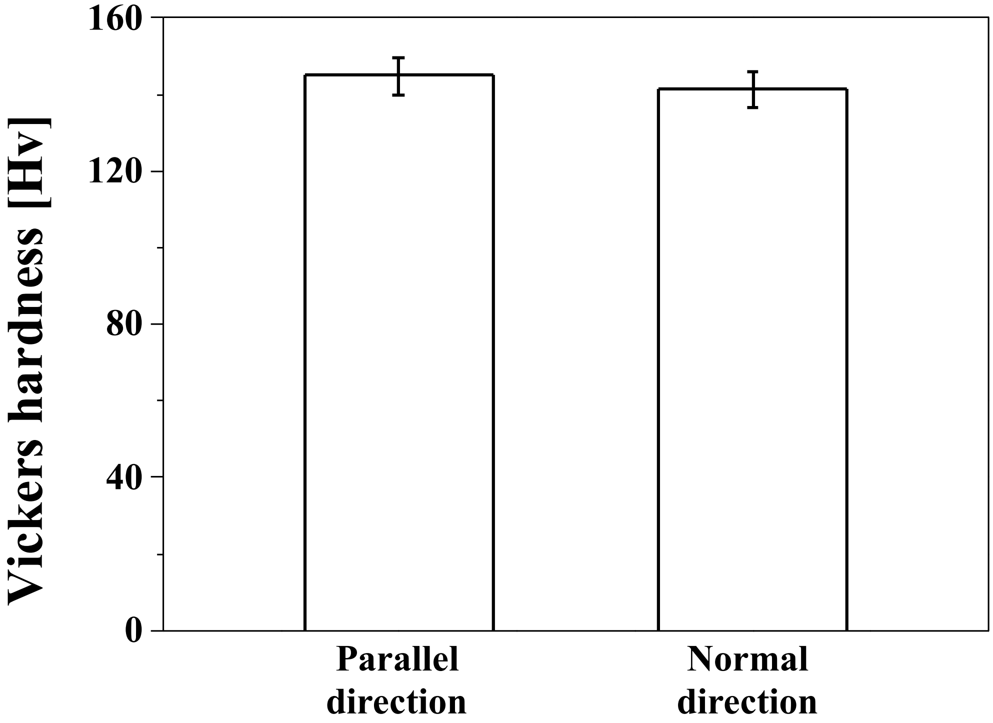

Supplementary Fig. S6 Vickers hardness for Al/BNNS composite measured on parallel and normal direction. The hardness measured parallel to the pressing direction is similar to that measured normal to the pressing direction. It means that the BNNS is dispersed in the Al matrix with random orientation.

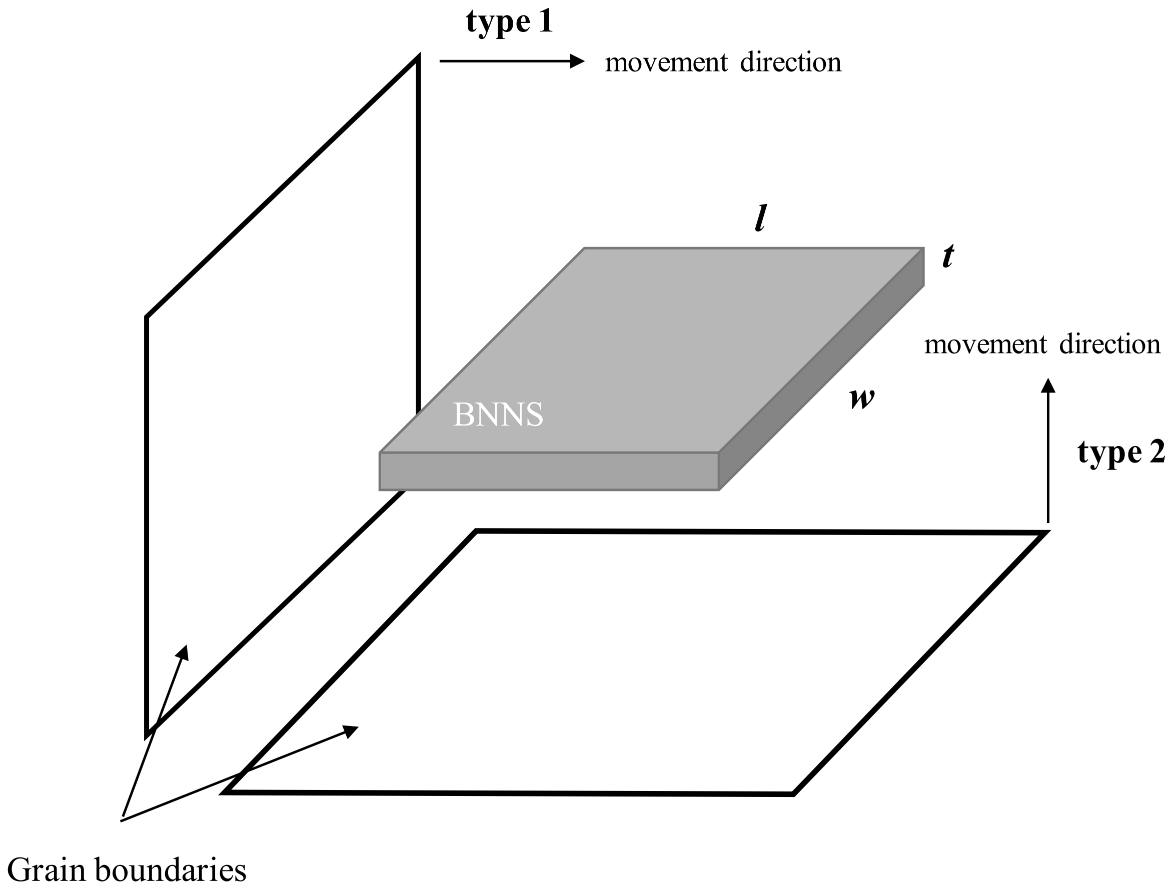

Supplementary Fig. S7 Schematic illustration of two types of direction for boundary movement involving plate-type particles with volume w_s (width) × t_s (thickness) × l_s (length). Types 1 and 2 represent grain boundary movement to the parallel and normal directions with respect to BNNS alignment, respectively

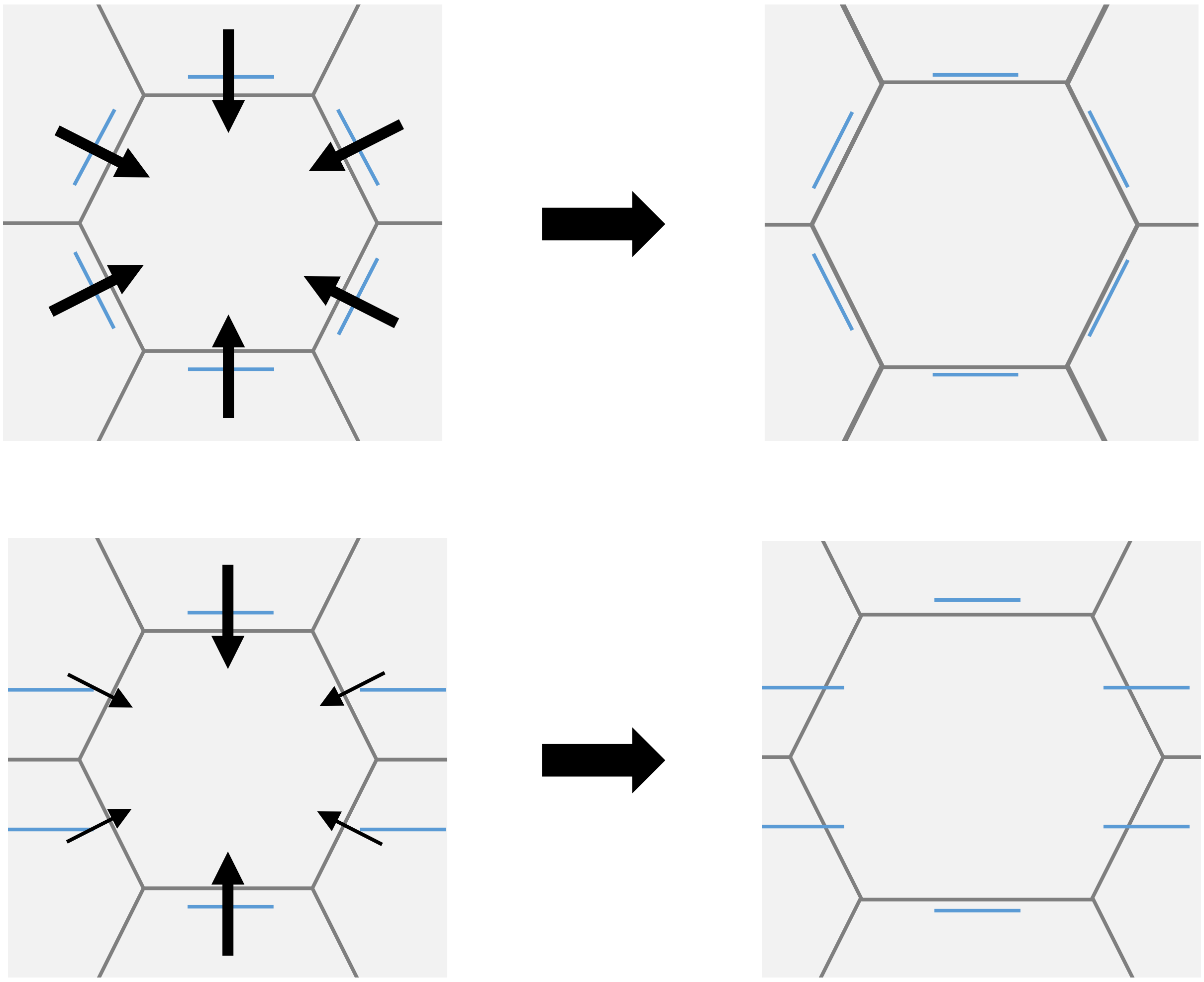

Supplementary Fig. S8 Schematic illustration of the grain growth behavior of randomly and unidirectionally oriented plate-like particle-containing composites. For the plate-like particles with random orientation with higher volume fractions, it is more likely that the grain boundary moves toward the normal direction of the particles.
